# A Critical Overview of the Use of Platelet-Rich Plasma in Equine Medicine Over the Last Decade

**DOI:** 10.3389/fvets.2021.641818

**Published:** 2021-03-31

**Authors:** Livia Camargo Garbin, Catalina Lopez, Jorge U. Carmona

**Affiliations:** ^1^Department of Veterinary Clinical Sciences, Faculty of Medical Sciences, School of Veterinary Medicine, The University of the West Indies at St. Augustine, St. Augustine, Trinidad and Tobago; ^2^Grupo de Investigación Terapia Regenerativa, Departamento de Salud Animal, Universidad de Caldas, Manizales, Colombia

**Keywords:** platelet-rich plasma, equine, sports medicine, musculoskeletal disease, orthopedics

## Abstract

In the 1990s, the role of platelets in inflammation and tissue healing was finally recognized. Since then, the clinical use of platelet-derived products (hemocomponents), such as, platelet-rich plasma (PRP), markedly increased. The promise of a more economical option of a disease-modifying treatment led to the intensive and continuous research of PRP products and to its widespread clinical use. A number of protocols and commercial kits have been developed with the intention of creating a more practical and reliable option for clinical use in equine patients. Still, the direct comparison between studies is particularly challenging due to the lack of standardization on the preparation methods and product composition. The incomplete reports on PRP cellular concentration and the poorly designed *in vivo* studies are additional matters that contest the clinical efficiency of this biomaterial. To overcome such challenges, several *in vitro* and *in vivo* studies have been proposed. Specifically, experiments have greatly focused in protocol optimization and its effect in different tissues. Additionally, *in vivo* studies have proposed different biological products envisioning the upgrade of the anti-inflammatory cytokines trusting to increase its anti-inflammatory effect. The individual variability and health status of the animal, type of tissue and condition treated, and protocol implemented are known to influence on the product's cell and cytokine composition. Such variability is a main clinical concern once it can potentially influence on PRP's therapeutic effects. Thus, lack of qualitative and quantitative evidence-based data supporting PRP's clinical use persists, despite of the numerous studies intended to accomplish this purpose. This narrative review aims to critically evaluate the main research published in the past decade and how it can potentially impact the clinical use of PRP.

## Introduction

The discovery of multiple growth factors (GFs) within platelet's alpha-granules in the 80's (1–3) led to the investigation of platelets as a potential “regenerative” treatment. The significant improvement obtained in reconstruction of large alveolar-mandibular defects in humans (4), and the successful healing of complicated wounds (5) increased the interest for use of platelet-derived products.

In veterinary medicine, the use of platelet-rich plasma (PRP) started in the field of sports medicine, for the treatment injuries in ligament and tendons (6–8), and it has been used for several veterinary surgical procedures as a biomaterial (9). PRP was used in horses with osteoarthrosis (10), based on previous positive outcomes in humans (11–14). However, controversial results obtained in experimental studies with PRP use in horses (15, 16) increased skepticism of the clinical efficiency of PRP. Positive results have been observed from studies with higher chance of bias (17), particularly, in equine studies when compared to human studies (17).

The variation in results obtained in treatments using PRP in both research and clinical scenarios are more likely to be related with its cellular and cytokine profile, as well as its diverse application methods (18). The PRP biologic profile depends on the preparation, which varies in different studies. Additionally, genetic and physiological variability of the equine patients or experimental subjects augments the inconsistency of PRP efficacy among individuals (19). For this reason, several methods of PRP preparation and commercial kits have been proposed for optimization of the product and to develop a more standardized and practical method of delivery and preparation. However, insufficient evidence-based data supporting its clinical use remains. The objective of this review was to critically evaluate the most relevant research published in the past decade and to suggest potential area for improvement in research. Additionally, this review evaluates, compares, and intend to adapt the human PRP classification systems to the methods available for preparation of PRP-products for equine practice.

## *In Vitro* Platelet-Rich Plasma Research

In horses, the outcomes with the use of PRP tends to be variable in *in vitro* experiments, depending on the scenario (20–22). *In vitro*, the variable effects of PRP can be easily observed depending on cellular content, volume and tissue tested (20–22). This variability can be particularly challenging when treating organs with different types of tissue, such as joints (23). In equine cartilage, higher concentrations of leukocyte and platelet-rich gel (L-PRG) supernatant produced more sustainable release of anabolic factors compared with pure platelet-rich gel (P-PRG) supernatant, under inflammatory conditions (21). Growth factors (GF) such as platelet-derived growth factor-BB (PDGF-BB), transforming growth factor-beta 1(TGF-β_1_), and anti-inflammatory cytokines as interleukin 1 receptor antagonist (IL-1Ra) were observed (21). The dynamic of cytokine release varied according to the cellular content of the platelet-rich gel (PRG) (21). In this case, it is challenging to know if such effects on GF release are due to the higher concentrations of leukocytes or platelets on PRP. Additionally, fibrin content could influence the dynamic of GF release (22, 24). In synovium on the other hand, both preparations of allogeneic PRP induced anabolic and anti-inflammatory effect, but leukocyte-reduced preparations increased gene expression of chondrogenic factors (22). In Gilbertie et al. (20) synoviocytes stimulated with LPS and treated with allogeneic platelet-rich lysate (PRP-L) protected chondrocytes challenged with synoviocyte-conditioned media. This effect correlated with an increase in anti-inflammatory cytokine compared to reduction in pro-inflammatory mediators in synoviocyte media (20).

In synovial cells suspended in fluid, PRP resulted in higher prostaglandin E 2 (PGE_2_) concentration but conversely, increased concentrations of IL-1Ra and decreased oxidative stress (15). It is important to note that the type of synovial cell though, was not well-defined. Another important factor frequently not discussed is the interaction of GFs in tissues which might lead to different outcomes. Previous studies (21, 22) suggest that leukocyte concentration within PRP affects cartilage and synovium differently. Once synovium interacts directly with cartilage and plays an important role in osteoarthritis (25), it may be prudent to avoid use of leukocyte-rich PRP in joints.

In normal equine tendon and ligament, platelet-derived products had a different release dynamic of bioactive factors (26). Equine normal suspensory ligament and tendon explants were cultured with different concentrations of leukocyte-reduced platelet-rich gel supernatant (Lr-PRGS) and leukocyte-reduced plasma supernatant (Lr-PL) (26). Overall lower concentrations of Lr-PRGS induced the most adequate pattern of anti-inflammatory factors in ligament and tendon explants when compared to other hemoderivatives (26). Additionally, leukocyte-reduced platelet products demonstrated more sustainable release of GFs for tendon and ligament explants compared to other platelet-lysate products (26).

In ligaments under inflammatory conditions though, both leukocyte-concentrate and leukocyte-reduce platelet products presented anti-inflammatory effects (27). These studies showed that the anti-inflammatory effects of platelet-derived products might relate to higher concentrations of PDGF-BB in these products (26, 27). Both studies showed a significant correlation between levels of PDGF-BB and IL-1Ra release by the tissues (26, 27). However, the authors did not show the direct correlation of the increase in IL-1Ra release and inhibition of IL-1 neither its downstream effects on tissue matrix. Further, research is necessary to confirm these assumptions.

## *In Vivo* Platelet-Rich Plasma Research

The clinical application of PRP has been more frequently used in the field of equine sports medicine. Particularly in tendons (28) and ligament injuries, application of PRP has been overall successful. In mechanically induced lesions of the superficial digital flexor tendon (SDFT), intralesional injections of PRP were performed 7 days after trauma (28). Treated tendons presented significantly more production of collagen, glycosaminoglycan and cellularity compared to controls (28). The use of PRP in this experiment also improved tissue organization and biomechanical properties compared to placebo (28). Tendons treated with PRP were more metabolically active and showed an advanced repair after 23 weeks (28). The improvement in collagenous matrix organization was also observed through computerized analysis of ultrasonographic images in a similar study (29). The authors believed that the earlier resolution of inflammation and the improved callus formation observed in PRP-treated tendons, resulted in better repair tissue in later stages of the healing process when compared to control (29).

In naturally occurring tendinopathies of the SDFT (30), PRP was injected up to 8 weeks after clinical onset. Lameness decreased significantly 8 weeks after treatment, while in control groups, this was observed at 12 weeks, although no difference in cross-sectional area was observed (30). Eighty percent of PRP-treated horses reached their previous levels of performance or higher, 12 months after treatment, while control horses reached similar level in 24 months (30).

Leukocyte-rich PRP demonstrated significant clinical improvement when used on sesamoiditis associated with suspensory ligament branch desmitis (31). Horses in this group were significantly more likely to start racing in a short-term after treatment compared to controls (31). However, there was no difference between groups when races were performed in a long-term after treatment (31).

In equine normal joints, platelet-derived product (Equine Platelet Enhancement Therapy, E-PET, Pall Corporation), activated with thrombin induced an inflammatory response, whereas, platelets activated with CaCl_2_ did not (32). The synovial fluid levels of PDGF and TGF-β_1_ were low after platelet application, thus authors believe the overall positive effects of this therapy may not be attributed to local changes in PDGF and TGF-β_1_ concentration (32). Furthermore, GF concentration increased significantly after contact with synovial fluid, which may indicate that exogenous activation of PRP may be unnecessary (32).

Concerning joint disease, variable results have been obtained in clinical improvement when platelet-related products are used for moderate to severe osteoarthritis (33). Although, PRP presented anti-oxidant effects in joints, it also increased PGE_2_ concentration and decreased quality of hyaluronic acid in synovial fluid after 48 h of treatment (15). For this reason, the authors suggested caution with the use of PRP is osteochondritis dissecans, immediately after surgery (15).

In bone healing, PRP increased bone consolidation one month after injection in a comminuted diaphyseal fracture of the tibia in a donkey. Delayed bone consolidation and a bone gap were observed 50 days after surgery, although the animal manifested good clinical condition. Authors concluded that PRP was effective as an adjuvant therapy in bone healing (34).

The interest in using this therapy in other systems increased due to PRP prevalent use in sports medicine. In skin wounds, controversial results have been observed (16, 35). While this treatment did not influence on formation of collagen types I and III, during the healing process of wounds (35), in other studies PRP favored the formation of excessive granulation tissue, which significantly slowed wound healing up to 3 weeks after creation of the lesion (16). Controversially, in another study, PRP treatment did reduce healing time in wounds on the distal limb of horses, but only in 41.67% of the cases (36). It is known that second intention wound healing in horses may be more problematic compared to other species (8). The formation of exuberant granulation tissue delays healing, and is especially common in limb wounds where the lesions of the aforementioned studies were induced (37).

Promising results with the use of PRP were obtained in endometritis cases in mares (38, 39). Significant decrease in fluid and polymorphonuclear cell influx were observed in mares with different levels of endometritis treated with PRP 4 h after artificial insemination, compared with cycles which the mares were untreated (38). When applied either before or after artificial insemination, PRP also reduced polymorphonuclear cell infiltrate and cyclo-oxygenase 2 (COX-2) labelled cell compared with control mares (38, 39). Overall, the authors believed PRP reduced inflammatory response in mares with persistent mating-induced endometritis (38, 39).

The anti-inflammatory effect of PRP seems to rely in different mechanisms that are slowly being clarified. Platelet-rich plasma was able to inhibit nuclear factor Kappa B (NF-κB) (40, 41) a key pathway activated during the inflammatory process (42, 43). Additionally, PRP reduced chemotaxis to polymorphonuclear cells due to the increase of lipoxin A4 and other chemokines (44–46). Such effects could result in downregulation of important proinflammatory mediators such as TNF-α, IL-1, and COX-2, observed in various inflammatory processes (47).

Inconsistency in results obtained in PRP research is a frequent concern. The type of cases and lesions being treated are highly inconsistent (48). There is also a lack of substantial information regarding the standardization and characterization of clinical protocols using PRP. Typically, when cell count is performed on in PRP products, only platelet and in some occasions white blood cell (WBC) numbers are reported. Insufficient characterization of WBC within PRP was observed in previous studies (28, 30). It is well-known that the types of leukocytes (i.e., neutrophil) can potentially lead to an increase in pro-inflammatory response (49). Finally, when describing cytokine composition of PRP and growth factor content, authors frequently report PDGF and TGF-β_1_. Although, high concentrations of TGF- β_1_ (1, 2, 19, 21) and PDGF (19, 21, 50) are found in PRP, other growth factors and cytokines are also abundant (21, 51–53), and are not frequently studied despite their role in tissue physiology.

The activity or use of the animals and low number of horses are additional factors increasing variability (30, 38, 39) in PRP research. Lack of proper control groups or standard therapeutic comparisons are other pitfalls frequently observed in clinical studies with the use of PRP (33). Based on such issues, different alternatives have been proposed in research. Studies proposed to compare the treated animal to itself before and after treatment (54). Another alternative was to perform analgesia on the affected limb (peripheral nerve block) and compare the treated horse to itself before and after analgesia (33). Finally, it was proposed to compare the treated horses to other animals of same age, sex, activity, and athlete performance as controls (48). The short duration of the studies, not allowing proper time for tissue repair was another pitfall identified (55). Better research practices in PRP research are paramount for obtaining reliable results that support clinical use of PRP.

It is imperative to analyze some aspects related with PRP-derived products before taking into consideration the different methods that aim to produce PRP. Below, the classification of PRP in the human regenerative medicine field, as well as the methods used for preparation of PRP in equine practice will be revised.

## Classification of Platelet Concentrates in Human Regenerative Medicine

An intense debate for a consensus definition and classification of the plethora of PRP-related products of clinical use persists in the scientific community in the past decade (8, 56–62). From Mishra et al. (63) to Kon et al. (64), different classifications methods have been proposed for human PRP. Although PRP has been widely used in veterinary medicine, no valid standardized classification methods for platelet-derived products were proposed. The increase use of PRP by veterinarians showed the urge to stablish important aspects of PRP preparation and application (58, 59, 65). A standard classification system and procedures for its application in veterinary practice shall improve comparison among clinical studies evaluating the efficacy of PRP. To note, a recent study of PRP in humans found that only 10% of the studies provided detailed information of PRP preparation methods (65). Additionally, only 16% of the studies presented quantitative analysis on the composition of PRP (65).

Currently, there are several classification systems for human PRP-related products. These classifications include the description of platelets and leukocytes (including the type and number of leukocyte subpopulations), activation methods, and the final volume of plasma in PRP (65). In general, it can be concluded that these PRP classification systems are useful to document effectively the PRP used in experimental and clinical situations. These methods aim to segregate what is the ideal PRP-hemocomponent, for a particular tissue, in specific clinical scenarios (62, 64). A comprehensive synopsis of the most relevant classification proposals for PRP-related products in human regenerative medicine is presented in Table 1.

**Table 1:** Systems proposed for platelet-rich plasma classification in human regenerative medicine (2010–2020).

**Authors**	**Variables considered and additional commentaries**	**Classification system**
DeLong et al. (59)	PAW system • Platelet (PLT) concentration (μL) • Method (exogenous or endogenous) of PLT activation (Ac) • Presence or absence of white blood cells (WBCs): including neutrophils.	PLT concentration • P1: ≤ to baseline PLT concentrations • P2: >baseline concentrations to 750 × 10^3^ PLT/μL • P3: >750 to 1,250 × 10^3^ PLT/μL • P4: >1,250 × 10^3^ PLT/μL.
		Methods of PLT activation • Endogenously: no symbols • Exogenous activating (Ac): x.
		WBC • Above **(A)** to baseline • Below/equal to **(B)** baseline • Neutrophils: α (above) (below) is added blood levels
Mishra et al. (63)	Sports medicine PRP classification. • WBC concentration • Activation (Ac): (–) no activation, (+) activation • PLT concentration: A (5x or >blood levels), B <5x blood levels.	Type 1: WBC↑, Ac (–), A or B. Type 2: WBC↑, Ac (+), A or B. Type 3: WBC↓, Ac (–), A or B. Type 4: WBC↓, Ac (+), A or B.
Dohan Ehrenfest et al. (60)	Platelet concentrate classification. • WBC presence and concentration • Fibrin architecture. • Activation (Ac): intrinsic. • No anticoagulant.	Pure PRP (P-PRP) – or leukocyte-poor PRP: products without WBCs and low-density fibrin network after Ac. Leukocyte and PRP (L-PRP) products: preparations with leukocytes and with a low-density fibrin network after Ac. Pure platelet-rich fibrin (P-PRF) – or Leukocyte- Poor PRF: products without WBCs and high-density fibrin network. Leukocyte- and PRF (L-PRF): products with leukocytes and high-density fibrin web.
Mautner et al. (62)	PLRA classification • PLT/μL • WBC/μL (including neutrophils) • Red blood cells (RBCs) • Activation (A) • Volume of PRP (ml)	P:PLT count per μL
		L: WBC • (–): <1% of neutrophils in WBC • (+): >1% of neutrophils in WBC
		R: RBC/μL • (–): <1% of RBC in PRP • (+): >1% of RBC in PRP
		A: Activation (No = –, Yes = +)
		PRP injected volume (mL).
Magalon et al. (61)	DEPA classification • D: dose of injected PLTs/PRP-injected volume • E: % PLT recovered in the PRP. • P: purity of the PRP obtained (relative composition of PLTs, WBCs, and RBCs). • A: activation process.	D: • (a) Very high dose of injected platelets: >5 billion • (b) High dose of injected platelets: 3 to 5 billion • (c) Medium dose of injected platelets: f 1 to 3 billion • (d) Low dose of injected platelets: <1 billion.
		E: • (a) High efficiency: if the recovery rate in PLTs is >90% • (b) Medium efficiency: if the recovery rate in PLTs is between 70 and 90% • (c) Low efficiency: recovery rate of PLTs is between 30 and 70% • (d) Poor efficiency: recovery rate is <30%
		P: • (a) Very pure PRP: if the percentage of PLTs is >90% • (b) Pure PRP: between 0 and 90% of the PLTs • (c) Heterogeneous PRP: if the percentage of PLTs is between 30 and 70% • (d) Whole-blood PRP: if the percentage of PLTs in the PRP is <30% compared with RBCs and WBCs.
		A: • Ac used for activating PRP.
Kon et al. (65)	PRP coding system. • 6 digits grouped in pairs indicating parameters of PLT composition, purity and Ac: N_1_ N_2_ -N_3_ N_4_ -N_5_ N_6_.	N_1_ & N_2_ indicate the PLT concentration of PRP.
		N_3_ & N_4_ indicate the purity of the PRP, referring to the absence (0) or presence (1) of RBCs and the concentration of WBCs (0, 1, 2, 3.).
		N_5_ and N_6_ refer to the activation. • N_5_ indicates if activation is endogenous (0) or if PRP is activated before its injection (1). • N_6_ mentions the addition of calcium for activation (0 = no, 1 = yes).

*PLT, platelet; WBC, white blood cell; Activation, Ac*.

At this point, we made a search of experimental, hematological, and clinical equine PRP studies intending to found the specific concentration of each cell type of each evaluated PRP in order to classify these PRP-related products according to some human PRP classification (see Tables 2, 3).

**Table 2:** Semiautomated methods used for PRP production from equine blood and potential classification according to the systems proposed for human PRP classification.

**Authors**	**Type of study**	**Design**	**Type of PRP Kit and protocol**	**PLT, WBC, and RBC concentrations per μL of PRP**	Human PRP Classification^*^
Fontenot et al. (66)	Hematological study	Comparison between three manual and semiautomated methods for producing PRP	Genesis CS; Vet-Stem, Poway, CA, USA.	PLT × 10^3^/μL: 359.2 ± 201.9 WBC × 10^3^/μL: ND RBC × 10^6^/μL: NR	It is not possible to classify this PRP according to the published information.
Textor et al. (67)	Experimental *in vivo* study	Evaluation during time of joint response after PRP injection. *n = 7* horses	The Equine Platelet Enhancement TherapyTM (E-PET); Pall Corporation, Port Washington, NY, USA.	PLT × 103/μL: 542 ± 196.3 WBC × 103/μL: 13.1 ± 3.46 RBC × 106/μL: ND	L-PRP (61) P2A (60) Type 1B (64)
Garrett et al. (31)	Randomized clinical trial	Patients with proximal sesamoid bone inflammation and associated suspensory ligament branch desmitis were treated either with PRP or saline. *n =* 39	GPS II; Biomet, Warsaw, IN, USA g: Time:	PLT × 103/μL: 966 ± 189 WBC × 103/μL: NR RBC × 106/μL: NR	It was not possible to classify this PRP according to the published information.
Hessel et al. (68)	Hematological study	Were compared hematological variables of equine PRPs obtained with 4 commercially available systems and a non-commercial double-centrifugation technique. *n* = 6 horses	AngelTM; Arthex Inc., Naples, FL, USA. ACPTM (autologous conditioned plasma); Arthex Inc., Naples, FL, USA. E-PETTM; Pall Corporation, Port Washington, NY, USA. GPSTMIII (gravitational platelet separation system); Biomet Biologics Inc., Warsaw, IN, USA.	PLT × 103/μL: 320 ± 198 WBC × 103/μL: 9.1 ± 6.0 RBC × 106/μL: NR PLT × 103/μL: 183 ± 39.7 WBC × 103/μL: 0.6 ± 0.3 RBC × 106/μL: NR PLT × 103/μL: 533.3 ± 198.2 WBC × 103/μL: 11 ± 2.5 RBC × 106/μL: NR PLT × 103/μL: 761 ± 240 WBC × 103/μL: 40.6 ± 3.9 RBC × 106/μL: NR	L-PRP (61) P2A (60) Type 1B (64) P-PRP (61) P2B (60) Type 1B (64) L-PRP (61) P2A (60) Type 1B (64) L-PRP (61) P3A (60) Type 1B (64)
Geburek et al. (30)	Randomized prospective controlled clinical trial	Patients with naturally occurring tendinopathies of forelimb SDFTs were randomly assigned to the PRP-treated group or control group. *n=* 20.	Osteokine^®^ Orthogen, Düsseldorf, Germany	PLT × 103/μL: 892.37 ± 364.7 WBC × 103/μL: 14.1 ± 7.0 RBC × 106/μL: ND	L-PRP (61) P3A (60) Type 1A (64)
Hauschild et al. (69)	Hematological study	Were compared hematological variables of PRP obtained with two different kits.	ACP^TM^ (autologous conditioned plasma); Arthex Inc., Naples, FL, USA.	PLT × 103/μL: 204.25 ± 34.87 WBC × 103/μL: 0.57 ± 0.27 RBC × 106/μL: 0.04 ± 0.02	P-PRP (61) P1A (60) Type 1B (64)
			E-PET^TM^; Pall Corporation, Port Washington, NY, USA.	PLT × 103/μL: 327.8 ± 186.93 WBC × 103/μL: 12.12 ± 2.21 RBC × 106/μL: 4.34 ± 1.49	L-PRP (61) P2A (60) Type 1B (64)

*PLT, platelet; WBC, white blood cell; RBC, red blood cell*.

* *For acronym mean, please see Table 1*.

**Table 3:** Manual methods used for PRP production from equine blood and potential classification according to the systems proposed for human PRP classification.

**Authors**	**Type of study**	**Design**	**Tubes and other materials used for PRP protocol**	**PLT, WBC, and RBC concentrations per μL of PRP**	Human PRP Classification^*^
Fontenot et al. (66)	Hematological study	Comparison between two manual and semiautomated methods for producing PRP.	60-mL syringe containing 8 mL of ACD-A. 10-mL plain glass tubes. Centrifugation at 1200 g/3 min Centrifugation at 2000 g/3 min. 50 mL conical tube	PLT × 10^3^/μL: 267.5 ± 142.8 WBC × 10^3^/μL: ND RBC × 10^6^/μL: NR	It was no possible to classify these PRPs according to the published information.
				PLT × 10^3^/μL: 399.4 ± 157.1 WBC × 10^3^/μL: ND RBC × 10^6^/μL: NR	
				PLT × 10^3^/μL: 433.0 ± 126.9 WBC × 10^3^/μL: ND RBC × 10^6^/μL: NR	
Maciel et al. (70)	Experimental *in vivo* study	Evaluation of PRP in equine burns.	15 mL Falcon tubes containing 10% sodium citrate. Centrifugation at 300 g/10 min	PLT × 10^3^/μL: 723.0 ± 50 WBC × 10^3^/μL: NR RBC × 10^6^/μL: NR	It was no possible to classify this PRP according to the published information.
Giraldo et al. (19)	Hematological study	Evaluation of the effect of intrinsic factors on equine PRP. *n* = 40	8.5-mL ACD-A tubes that were centrifuged two times at 120 g/5 min and 240 g/5 min.	PLT × 10^3^/μL: 304.3 ± 43.9 WBC × 10^3^/μL: 4.3 ± 2.2 RBC × 10^6^/μL: 0.14 ± 0.1	P-PRP (61) P2B (60) Type 1B (64)
Hessel et al. (68)	Hematological study	Were compared hematological variables of equine PRPs obtained with 4 commercially available systems and a non-commercial double-centrifugation technique. *n* = 6 horses.	60-mL syringe containing 8 mL of ACD-A. 10-mL plain glass tubesCentrifugated two times at 300 g/10 min.	PLT × 10^3^/μL: 310.4 ± 164.5 WBC × 10^3^/μL: 18.2 ± 11.8 RBC × 10^6^/μL: NR	L-PRP (61) P2A (60) Type 1B (64)
Moraes et al. (71)	Experimental *in vivo* research	Evaluation of the effect of PRP in equine joints. *n* = 8 horses.	Tubes containing sodium citrate that were centrifugated two times at 150 g/5 min and 800 g/5 min.	PLT × 10^3^/μL: 423.0 ± NR WBC × 10^3^/μL: 8.36 ± NR RBC × 10^6^/μL: NR	L-PRP (61) P2A (60) Type 1B (64)

* *For acronym mean, please see Table 1*.

## Equine PRP-Related Hemocomponents

The first research papers related with PRP characterization and clinical use in horses were published over 17 years ago (72). The most significant research performed in the field have been published over the last decade. In general, PRP can be obtained through three methods: automated, semi-automated, and manual procedures (73). At this point, it is important to clarify that eventually PRP can be defined as a volume of plasma containing different concentrations of platelets and leukocytes. Platelet-rich plasma will always have a small quantity of an anticoagulant to prevent platelet activation and clot formation prior to its clinical use (60). On the other hand, platelet-rich fibrin (PRF) is a biomaterial obtained from centrifuged blood without any anticoagulant added (74). Platelet-rich fibrin is a firm platelet gel that can be used as a biomaterial in many surgical procedures, although it is not easily injected (60, 75).

To the knowledge of the authors, no studies involving the use of automated cell separators for the preparation of PRP in horses have been published in the past decade. It is important to mention that the first description of the use of these type of devices for PRP production was performed by Carter et al. (72). This PRP product was prepared by using 1L-bags with ACD-A as anticoagulant and a blood cell separator device (72).

With regard to the semi-automated methods, several devices or specialized kits have been described for producing equine PRP (66–69, 76). In general, these kits include a system for collecting blood from the patient, a sterile blood recipient for PRP production and a device for centrifugation (73). There is one method that does not require centrifugation in which PRP is obtained by gravity [E-PET^TM^ (67–69). Some of these kits have been validated and adjusted for equine blood, whereas others have been used with no modification of the original centrifugation protocol used for human blood (73). Table 2 presents a summary of the laboratory, *in vivo* and clinical studies performed in which PRP semiautomated kits were evaluated. There is a lack of substantial information related to cell composition of the PRP produced by semiautomated procedures. Consequently, proposing or to adapt human classification methods to equine PRP becomes challenging. It is important to highlight that only one paper presented a complete description of the platelets, leukocytes and red blood cells for PRP using two different semiautomated devices (69).

Several manual methods have been proposed for PRP elaboration from equine blood (19, 66, 68, 70, 77). In general, these protocols include one or two centrifugation steps (73). Firstly, blood can be collected directly in standard tubes containing anticoagulants (i.e., sodium citrate) or blood bags containing citrate dextrose solution A (ACD-A). Secondly, blood is easily placed in conical tubes and centrifuged for preparation of PRP. Table 3 presents the most frequent manual methods used clinically in horses in which a complete hematological information is provided.

## Controversial Aspects for Producing Equine Platelet-Derived Hemocomponents in Clinical and Field Conditions

Equine practitioners are frequently faced with several questions about the selection of the most adequate method or device for producing equine PRP. It is paramount to mention that a strict aseptic technique is necessary during the whole process. The main source for bacterial contamination during PRP preparations is; the skin of the patient, the flora from the clinician's hands and upper respiratory tract, and the environmental bacteria. Thus, the risks associated to potential bacterial PRP contamination or PRP-associated infections could be diminished by using a high standard sterile technique. Manipulating the blood in a dust-free environment with minimal air currents are important practices to augment safety for PRP application (78).

Equine practitioners are faced with selecting the most suitable method or device for obtaining PRP in their particular conditions. This election relies on several patient-associated factors, such as, the tissue or organ to be treated, lesion, breed, gender, age, and health status (19). Among the extrinsic factors, financial restraints, the availability of equipment, and adequacy of the facility should be considered.

In general, manual methods intended for PRP production present lower cost, but are time-consuming and require a high-quality centrifuge. Semiautomated kits on the other hand are regularly expensive but frequently more practical. The decision of the methods to use will depend on the extrinsic factors discussed and on the PRP demand. For clinicians that work in clinics and do have a significant demand for PRP treatment, it might be profitable in a long term to acquire a high-precision centrifuge. In cases where clinicians work mainly in the field, it would be more indicated to use semiautomated kits or even gravity-filtration systems (E-PET/V-PET). Other alternative would be to simply purchase the platelet products from laboratories that provide such services.

## Final Considerations

Several *in vivo* and *in vitro* experiments have shown beneficial effects of platelet-related products in modulation of the inflammatory process and tissue healing. To optimize PRP composition and facilitate its preparation, numerous methods have been described. Although, the literature in PRP is extensive, lack of evidence and consensus about what method or PRP composition to use in different disease or tissue trauma remains.

Although, the literature in equine PRP is vast, standard information about PRP composition, activation, and application methods are frequently lacking. This limitation prevents or limits the adaption of the classification methods used for PRP in humans to be used in equine platelet products. At this point, the creation of a particular system for PRP classification in horses is of paramount importance to improve, refine, and address new basic and clinical research in this growing research field.

A more detailed information about the methods of preparation, application and composition of the products used in equine research should be encouraged and strongly asked to be included for new equine PRP studies.

## Author Contributions

All the authors listed have made a substantial and direct intellectual contribution to the work. All authors have read and approved the manuscript for publication.

## Conflict of Interest

The authors declare that the research was conducted in the absence of any commercial or financial relationships that could be construed as a potential conflict of interest.
